# Impacts of Brain Serotonin Deficiency following *Tph2* Inactivation on Development and Raphe Neuron Serotonergic Specification

**DOI:** 10.1371/journal.pone.0043157

**Published:** 2012-08-17

**Authors:** Lise Gutknecht, Naozumi Araragi, Sören Merker, Jonas Waider, Frank M. J. Sommerlandt, Boris Mlinar, Gilda Baccini, Ute Mayer, Florian Proft, Michel Hamon, Angelika G. Schmitt, Renato Corradetti, Laurence Lanfumey, Klaus-Peter Lesch

**Affiliations:** 1 Molecular Psychiatry, Laboratory of Translational Neuroscience, Department of Psychiatry, Psychosomatics and Psychotherapy, University of Wuerzburg, Wuerzburg, Germany; 2 Department of Preclinical and Clinical Pharmacology, University of Florence, Florence, Italy; 3 Department of Psychiatry, Psychosomatics and Psychotherapy, University of Wuerzburg, Wuerzburg, Germany; 4 Center of Psychiatry and Neuroscience, National Institute for Health and Medical Research (INSERM U894), Medical Faculty Pierre and Marie Curie, Paris, France; INSERM, UMR-S747, France

## Abstract

Brain serotonin (5-HT) is implicated in a wide range of functions from basic physiological mechanisms to complex behaviors, including neuropsychiatric conditions, as well as in developmental processes. Increasing evidence links 5-HT signaling alterations during development to emotional dysregulation and psychopathology in adult age. To further analyze the importance of brain 5-HT in somatic and brain development and function, and more specifically differentiation and specification of the serotonergic system itself, we generated a mouse model with brain-specific 5-HT deficiency resulting from a genetically driven constitutive inactivation of neuronal tryptophan hydroxylase-2 (Tph2). *Tph2* inactivation (*Tph2*−/−) resulted in brain 5-HT deficiency leading to growth retardation and persistent leanness, whereas a sex- and age-dependent increase in body weight was observed in *Tph2*+/− mice. The conserved expression pattern of the 5-HT neuron-specific markers (except Tph2 and 5-HT) demonstrates that brain 5-HT synthesis is not a prerequisite for the proliferation, differentiation and survival of raphe neurons subjected to the developmental program of serotonergic specification. Furthermore, although these neurons are unable to synthesize 5-HT from the precursor tryptophan, they still display electrophysiological properties characteristic of 5-HT neurons. Moreover, 5-HT deficiency induces an up-regulation of 5-HT_1A_ and 5-HT_1B_ receptors across brain regions as well as a reduction of norepinephrine concentrations accompanied by a reduced number of noradrenergic neurons. Together, our results characterize developmental, neurochemical, neurobiological and electrophysiological consequences of brain-specific 5-HT deficiency, reveal a dual dose-dependent role of 5-HT in body weight regulation and show that differentiation of serotonergic neuron phenotype is independent from endogenous 5-HT synthesis.

## Introduction

Serotonin (5-hydroxytryptamine, 5-HT), a neuromodulator and neurotransmitter extensively distributed in the brain, is involved in the regulation of a wide range of basic physiological functions including developmental processes, synaptic plasticity as well as metabolic homeostasis, neuroendocrine function, appetite, energy expenditure, respiratory rate or sleep. In addition, the 5-HT system, also through its capacity to modulate the activity of other neuronal networks, shapes and regulates cognition and complex emotional behaviors including in interaction with environmental stressors (Gutknecht *et al*., unpublished data). It has been implicated in a wide spectrum of human behavioral traits as well as neurodevelopmental and neuropsychiatric disorders. An increasing body of evidence links 5-HT signaling alterations in early development to cognitive deficits, emotional dysregulation, and psychopathology in adult age [1], [2]. During ontogeny, 5-HT appears long before maturation of the raphe serotonergic neurons, suggesting a fundamental role in embryonic and brain development. Several *in vitro* and *in vivo* studies showed a morphogenetic effect of 5-HT on proliferation, migration, differentiation, connectivity and survival of neural cells, including the autoregulation of the development of the 5-HT system itself (reviewed in [3], [4]). To further analyze the significance of brain 5-HT in general development, the development and function of the brain and more specifically on the differentiation and specification of the serotonergic system itself, we have generated a mouse model displaying a brain-specific 5-HT deficiency resulting from a genetically driven inactivation of neuronal tryptophan hydroxylase-2 (Tph2, NCBI: protein, NP_775567.2; gene ID, 216343, [5]). Tph2 is the key enzyme in the synthesis of neuronal 5-HT [5]–[8] and catalyzes the hydroxylation of tryptophan (Trp) to 5-hydroxytryptophan (5-HTP) which is transformed to 5-HT by the amino acid decarboxylase (AADC). Tph2 is specifically expressed in the 5-HT neurons of the brainstem raphe complex and is exclusively responsible for the 5-HT synthesis within the brain [7], while Tph1 (NCBI: NP_033440) is the peripheral isoform. Tph2 null mutant (*Tph2*−/−) mice thus lack the ability to synthesize 5-HT specifically in brain and as a consequence have lost the capacity to release 5-HT and to establish serotonergic neurotransmission, while their peripheral 5-HT production is left intact.

Other but different models of genetically driven central 5-HT reduction were previously generated, such as the Tph2 R439H knockin mice [9], yet, this mutation only induces a 50% reduction of extracellular 5-HT in brain regions [10]. Mice with inactivation of the *Pet1* [11] and *Lmx1b* [12], [13] genes, coding for transcription factors involved in the specification of serotonergic neurons were also generated. However, both represent modification “upstream” of the specification process rather than a specific inactivation of neuronal 5-HT synthesis. In *Pet1* knockout mice (*Pet1* KO), 5-HT deficiency is incomplete with approximately 30% of the differentiated 5-HT neurons remaining in various raphe nuclei [14]. In conditional *Lmx1b* knockout mice (*Lmx1b* cKO), in which the gene deletion is driven specifically in serotonergic neurons, 5-HT neurons are generated but fail to differentiate and survive [15]. In contrast, in *Tph2*−/− mice, serotonergic neurons and their projections are still present but devoid of 5-HT [5].

In the present study, we investigated the impact of brain 5-HT deficiency on general and brain development, function of other monoamine neurotransmitters and on the specification and maintenance of the serotonergic system itself with focus on the neurochemical, molecular, cellular, and electrophysiological phenotype.

## Results

### Growth Retardation and Persistent Leanness in *Tph2*−/− but Age- and Sex-dependent Overweight in *Tph2*+/− Mice

**Figure 1:**
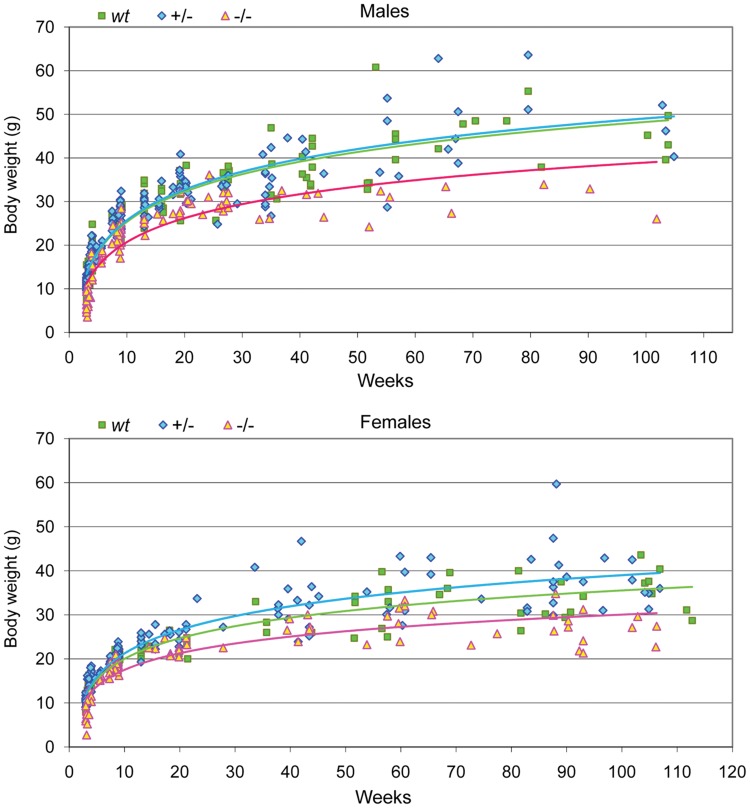
Growth and body weight during lifespan. Different male and female mice were weighted at different ages between 3 weeks and 2.2 years (421 males: *wt*, n = 155; +/−, n = 173; −/−, n = 93; and 374 females: *wt,* n = 147; +/−, n = 146; −/−, n = 81). *Tph2*−/− of both sexes displayed a growth retardation which persists throughout the lifespan as assessed by a constant significantly lower body weight than *wt* (males, p<0.001; females, p = 0.02) and +/− mice (males, p<0.001; females, p = 0.007). A significant sex-specific overweight was observed in *Tph2*+/− females from 24 weeks of age onward (+/− > *wt*, p = 0.031; +/− > −/−, p<0.001), while +/− males did not differ from *wt*. Age was used as covariable in the ANOVA analyses followed by Bonferroni-corrected pair-wise comparisons.

5-HT is implicated in the regulation of various physiological pathways influencing somatic growth, appetite, energy expenditure and storage. To evaluate the effect of central 5-HT deficiency on the regulation of these mechanisms, body weight was determined in different *Tph2* mutants compared to wildtype (*wt*) littermates at different ages from 3 weeks up to 2.2 years. First, as visible in Fig. 1, adult Tph2-deficient mice display an overall normal life expectancy. Using age as a covariable, growth retardation and leanness which persists throughout the lifespan was observed in *Tph2*−/− males (F_(2,417)_ = 11.56, p<0.001; Bonferroni-corrected pair-wise comparisons: −/− < *wt* and +/−, p<0.001) and *Tph2*−/− females (F_(2,370)_ = 14.624, p<0.001; −/− < *wt*, p = 0.02; −/− < +/−, p<0.001). During their first 24 weeks of life, *Tph2*−/− females had lower body weight than *wt* and +/− (F_(2,264)_ = 11.86, p<0.001, −/− < *wt* and +/−, p<0.001) but *wt* and *Tph2*+/− mice did not differ (p = 0.25). However, from 24 weeks of age onward, female +/− mice started to diverge from *wt* littermates showing an increase in their rate of weight gain (F_(2,88)_ = 15.95, p<0.001; *wt* < +/−, p = 0.031; −/− < *wt*, p = 0.013; −/− < +/−, p<0.001). Dissection revealed that *Tph2*+/− females can have impressive amount of fat stored in their abdominal and pericardial cavity, particularly in advanced age, while fat pads in *Tph2*−/− were much reduced compared to their littermates. Although *Tph2*+/− males also appeared to be more obese, their body weight did not significantly differ from *wt* controls before (F_(2,320)_ = 24.713, p<0.001; *wt vs* +/−, p = 1; −/− < *wt*, p<0.001; −/− < +/−, p<0.001) or after 24 weeks of age (F_(2,79)_ = 15.68, p<0.001; *wt vs* +/−, p = 0.46; −/− < *wt*, p<0.001; −/− < +/−, p<0.001). These results reveal a dual impact of central 5-HT in the regulation of somatic development and metabolic homeostasis and that brain 5-HT deficiency dose-dependently affects body weight via partially opposing mechanisms.

### Tph2 Inactivation Results in Brain 5-HT Deficiency and Reduction of Norepinephrine

**Figure 2:**
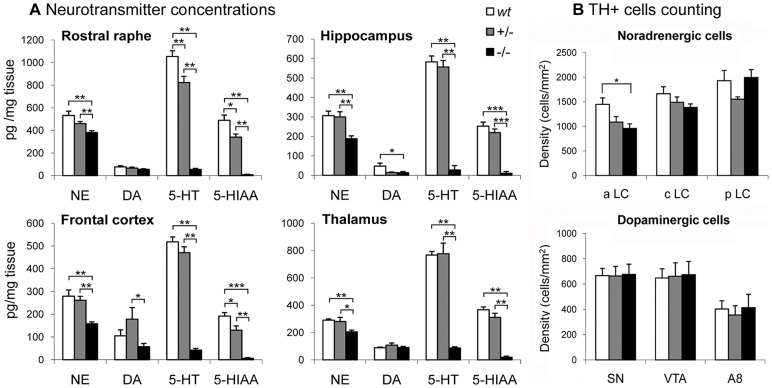
Neurotransmitter concentrations and TH positive cells counting in different brain regions. (***A***) HPLC analysis of norepinephrine (NE), dopamine (DA), serotonin (5-HT) and its metabolite 5-hydroxyindoleacetic acid (5-HIAA) in four different brain regions showed a drastic reduction of 5-HT and 5-HIAA concentrations in the brain of *Tph2*−/− compared to *wt* and +/− mice (n = 8). This reduction was further increased by an improved perfusion protocol (see corresponding results section) with e.g. 98.9% reduction in rostral raphe. A general reduction of NE concentration was also observed across brain regions of *Tph2*−/− mice compared to *wt* and +/− and a reduction of DA concentration in the hippocampus compared to *wt* mice. Kruskall-Wallis followed by Mann-Whitney-U-test: *p<0.05, **p<0.01, ***p<0.001. (***B***) Top: density of tyrosine hydroxylase (TH) positive cells in the locus coeruleus (LC, main noradrenergic cell cluster in the brain) showing a reduction of cell number in the anterior LC (aLC) of *Tph2*−/− mice (n = 8). Botom: density of TH positive cells in the main dopaminergic cell clusters: subtantia nigra (SN), ventral tegmental area (VTA) and retrorubal field A8 (n = 8). cLC: central LC, pLC: posterior LC. ANOVA followed by Tuckey-HSD: *p<0.05. Data are presented as means ± sem.

To assess the effect of Tph2 inactivation on brain 5-HT and its influence on the function of other neurotransmitter systems, monoamine concentrations were first analyzed by high-performance liquid chromatography (HPLC) in different brain regions of phosphate buffered saline (PBS) perfused animals (Fig. 2*A*).

#### 5-HT

Tph2 inactivation dramatically decreased 5-HT concentrations in all brain regions (H_(2)_>15.4, p<0.001). When compared to *wt* littermates, *Tph2−/−* mice exhibited a reduction of 5-HT concentrations reaching 94.8% in rostral raphe (RR), 95.2% in hippocampus (Hip), 91.8% in frontal cortex (FC) and 88.6% in thalamus (T) (all p<0.001). 5-HT in *Tph2+/−* mice was reduced to a much lesser extent and the only brain region for which the difference reached significance was the RR with a 21.8% reduction (p = 0.006) compared to *wt* mice.

#### 5-hydroxyindoleacetic acid (5-HIAA)

Levels of 5-HIAA, the main 5-HT metabolite, were different across genotypes in all regions (H_(2)_>16.2, p<0.001). The extent of reduction in *Tph2−/−* mice compared to *wt* was even more pronounced than for 5-HT with 98.4% reduction in RR, 96.0% in Hip, 96.7% in FC and 94.8% in T (all p≤0.001). In *Tph2+/−*, 5-HIAA concentrations were significantly lower in RR (−30.4%, p = 0.016), FC (−32.3%, p = 0.036) and tended to be decreased in T (−15.1%, p = 0.093) but not in Hip suggesting a region- and gene dose-dependent compensatory reduction of 5-HT turnover.

Because brain 5-HT was extremely reduced but not completely absent in *Tph2*−/− mice, we hypothesized that trace 5-HT may derive from platelets containing high levels of 5-HT and remaining in brain capillaries. Therefore, we carried out another 5-HT concentration analysis in a second set of animals which underwent a refined and more efficient perfusion protocol resulting in a more complete removal of residual blood from the brain. This analysis demonstrated a further reduction of 5-HT up to an additional 7.7% in *Tph2−/−* mice. Eventually, the 5-HT reduction in Tph2-deficient mice was 98.9% in RR, 96.2% in Hip, 94.0% in FC and 96.3% in T, strongly supporting the notion that most of the 5-HT traces detected in *Tph2−/− b*rain is carried by vascular perfusion and thus of peripheral origin.

#### Norepinephrine (NE)

5-HT deficiency in *Tph2−/−* mice was accompanied by a reduction of NE concentrations across brain regions (H_(2)_>9.7, p≤0.008). Compared to *wt* controls, *Tph2*−/− mice displayed significant reduction of 28.3% in RR (p = 0.005), 38.6% in Hip (p = 0.002), 43.3% in FC (p = 0.001) and 29.5% in T (p = 0.002). No such significant effect was observed in +/− animals.

#### Dopamine (DA)

Less consistent reductions were observed for DA levels with a genotype effect solely in Hip (H_(2)_ = 7.15, p = 0.028) and FC (H_(2)_ = 7.07, p = 0.029). In Hip, DA levels were decreased in *Tph2−/−* compared to *wt* mice (−71.9%, p = 0.019). In FC, *Tph2−/−* mice also exhibited significantly lower concentrations but only when compared to *Tph2*+/− mice (−67.7%, p = 0.010).

### Reduced Number of Noradrenergic Neurons in *Tph2−/−* Mice

To assess the effect of 5-HT deficiency on development and integrity of other neurotransmitter systems and to assess whether the reduction in NE and DA levels is due to a decreased number of catecholamine-specific neurons, noradrenergic and dopaminergic neurons were identified by tyrosine hydroxylase (TH) immunostaining and quantified in their respective nuclei (Fig. 2*B*). TH positive-cells were counted in the locus coeruleus (LC), the main central NE cell cluster, located in the brain stem as well as in the major dopaminergic nuclei, the substantia nigra (SN), ventral tegmental area (VTA) and A8 which, in its caudal boundary, is anatomically overlapping with the most rostral part of the dorsal raphe (DR). Counting of noradrenergic neurons in the LC revealed a significant reduction of cell densities in *Tph2*−/− mice in subparts of this cluster, rather than in the structure as a whole. Brain slices comprising anterior parts of LC exhibited a significant genotype effect (F_(2,17)_ = 5.23, p = 0.017) with a reduction of 33.8% (p = 0.015) in *Tph2−/−* and 24.9% (p = 0.081) in *Tph2*+/− mice compared to *wt* littermates. The difference between +/− and −/− groups was 11.9% without reaching significance (p = 0.668). In central LC, although *Tph2−/−* mice displayed a 17.5% reduction of cell density, the genotype effect was not significant (F_(2,23)_ = 1.63, p = 0.219). The posterior LC did not reveal a significant genotype effect (F_(2,19)_ = 2.43, p = 0.115). In the DA cell clusters SN, VTA and A8, no significant inter-genotypic difference was found. A detailed analysis within each cluster in a rostro-caudal dissection did also not show any difference. Since the volume of the cell clusters did not differ between genotypes in any of the analyzed structures, the cell density directly reflects the total number of TH-positive cells. The reduced NE brain concentration elicited by HPLC may then, at least in part, be explained by a reduction of the number of TH-positive noradrenergic neurons in the LC as a source of NE in terminal brain regions.

### Serotonergic Molecular Phenotype of Raphe Neurons Devoid of 5-HT is Conserved

**Figure 3:**
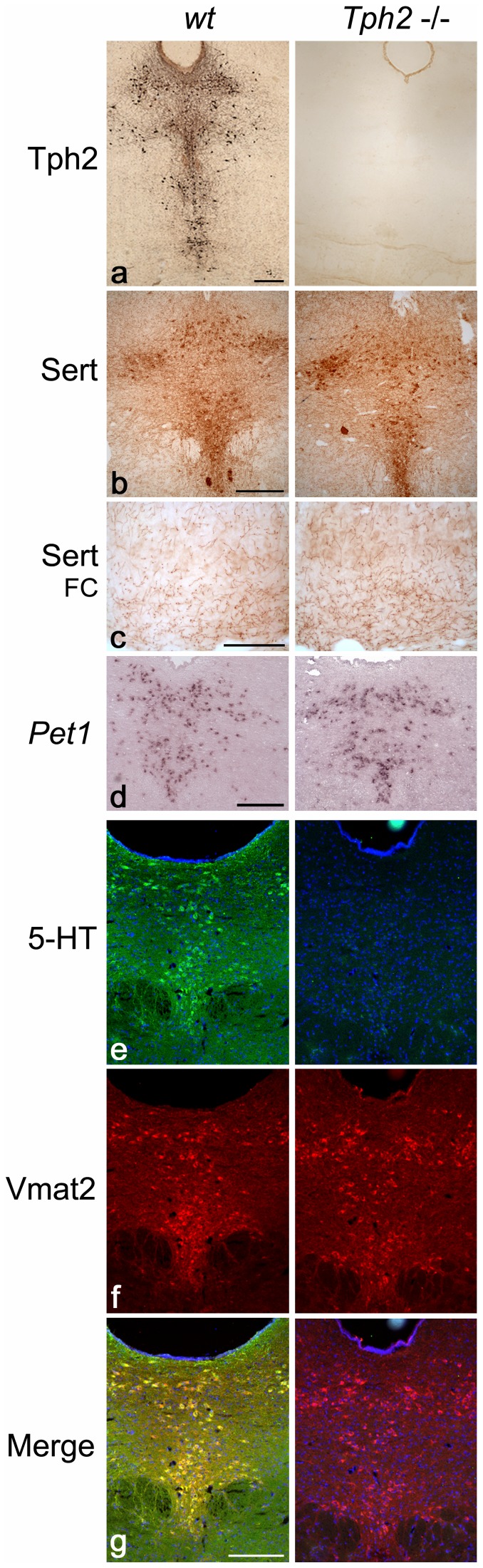
Histological characterization of serotonergic neurons. Detection of serotonergic-specific markers was performed on coronal brain sections of adult *wt* control (left panel) and *Tph2*−/− mice (right panel). Protein labeling was obtained by light immunohistochemistry (*a-c*) and immunohistofluorescence (*e-g*). (*a*) Labeling of Tph2 demonstrated its complete absence in the raphe of *Tph2*−/− mice. (*b*) The serotonin transporter (Sert) could be detected in both *wt* and *Tph2*−/− mice, in the raphe as well as along fibers in projection areas, e.g. in the frontal cortex (FC) as shown in (*c*). (*d*) Detection of the serotonergic-specific transcription factor *Pet1* in the raphe by *in situ* hybridization occurred similarly in *wt* and *Tph2*−/− mice. (*e*) Detection of serotonin (5-HT) in the raphe showed the absence of specific 5-HT immunoreactivity in *Tph2*−/− mice. Cell nuclei were also labeled by DAPI staining. (*f*) The vesicular monoamine transporter-2 (Vmat2) could be detected similarly in the raphe of both *wt* and *Tph2*−/− mice. (*g*) Merged images from (*e-f*) showed the colocalization of 5-HT and Vmat2 in the serotonergic neurons of *wt* (yellow in *g*) while *Tph2*−/− neurons were only labeled with Vmat2 (red in *g*). Taken together these results demonstrate that despite 5-HT synthesis deficiency, serotonergic neurons of *Tph2*−/− mice can develop and be maintained. Moreover, except Tph2 and 5-HT, they possess all known 5-HT-specific markers showing that their serotonergic specification took place. Bars represent 100 µm in (*c*) and 200 µm in (*a*), (*b*), (*d*), (*e-g*).

Given the known neurotrophic role of 5-HT in brain development, we have also investigated the effect of 5-HT synthesis incapacity on the development and differentiation of the serotonergic neurons themselves. Fig. 3a shows the absence of Tph2 immunoreactivity in *Tph2*−/− rostral raphe nuclei. It was also absent in other raphe nuclei and any further brain regions. We had previously shown the absence of 5-HT and Tph1 immunoreactivity in the brain of *Tph2*−/− mice ([5] and [7] respectively) by chromogenic immunohistochemistry. Here we additionally tested the other 5-HT specific markers. The 5-HT transporter (Sert) was normally present on the plasmatic soma membrane (Fig. 3b) as well as along projecting serotonergic fibers, e.g. in FC (Fig. 3c) but also in the other brain regions, such as Hip [5]. Immunofluorescent labeling confirmed the absence of specific 5-HT immunoreactivity in the raphe neurons (Fig. 3e) and other brain regions of *Tph2*−/− mice. The vesicular monoamine transporter-2 (Vmat2), which is in the raphe nuclei specifically expressed in *wt* 5-HT positive neurons (Fig. 3g), was also present in raphe neurons of *Tph2*−/− mice (Fig. 3f). Furthermore, using *in situ* hybridization, we demonstrated the maintained expression of the 5-HT neuron specific transcription factor *Pet1* in the raphe of *Tph2*−/− mice (Fig. 3d). These results, together with 5-HT_1A_ and 5-HT_1B_ autoradiography in raphe (see below), demonstrate a serotonergic-like phenotype and apparently normal cellular and morphological differentiation of the neurons despite the absence of Tph2 and endogenous 5-HT synthesis.

### Increased 5-HT_1A_ and 5-HT_1B_ Receptor Density and Stimulated [^35^S]GTP-γ-S Binding

**Figure 4:**
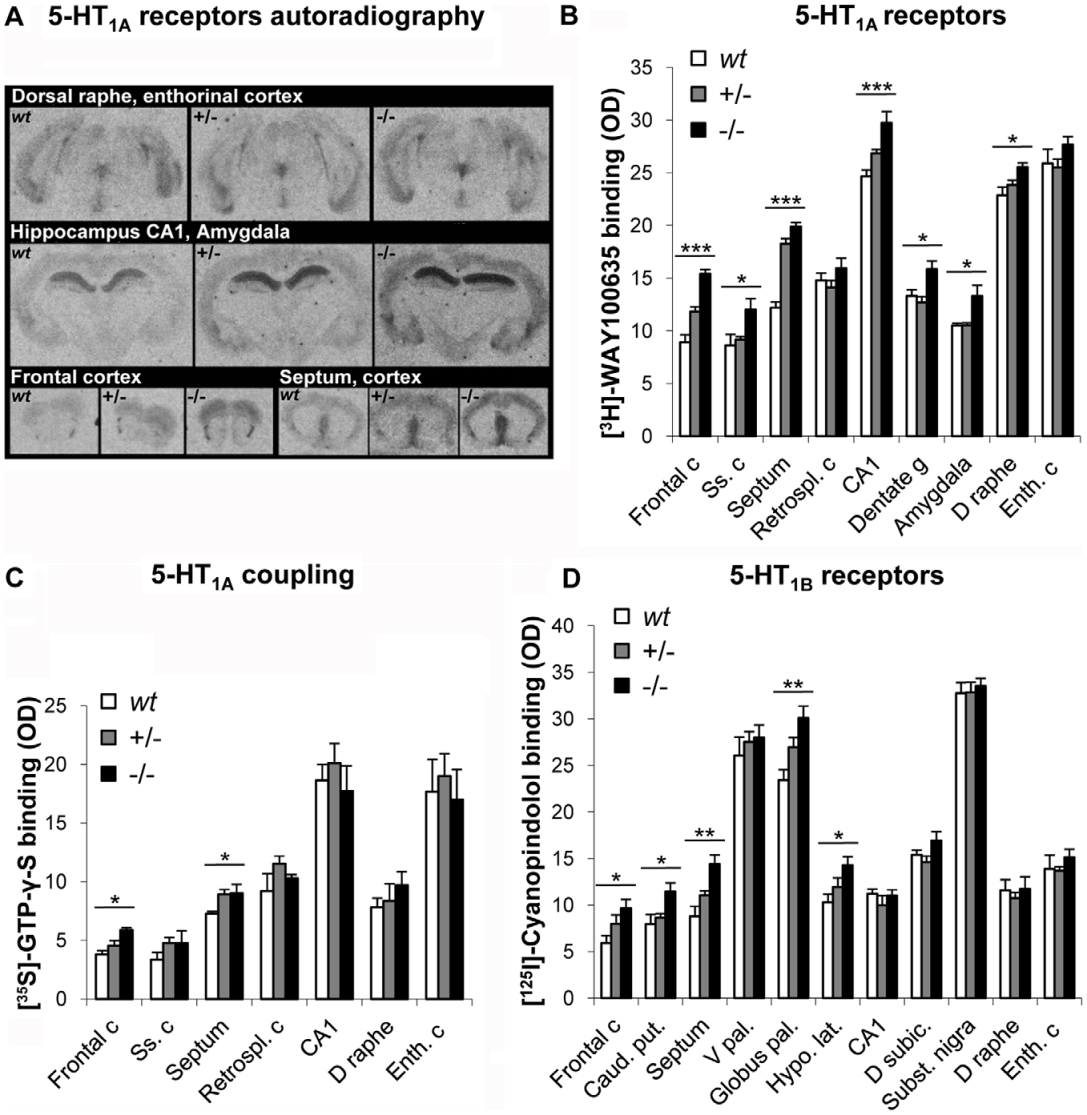
Quantitative autoradiography of 5-HT_1A_ and 5-HT_1B_ receptors in various brain regions. (***A***) Representative photomicrographs of autoradiograms following the binding of [^3^H]WAY100635 to 5-HT_1A_ receptors on whole coronal sections. The signal was visibly increased in e.g. the dorsal raphe, CA1 of hippocampus, frontal cortex and septum of *Tph2*−/− mice. (***B***) Binding density of 5-HT_1A_ receptors labeled by the radioligand [^3^H]WAY100635 was up-regulated in most of the brain regions of *Tph2*−/− mice. (***C***) 5-HT_1A_ receptor-mediated increase in [^35^S]GTP-γ-S binding after stimulation revealed enhanced 5-HT_1A_ coupling in the frontal cortex and septum of *Tph2*−/− mice. (***D***) Binding density of 5-HT_1B_ receptors labeled by the radioligand [^125^I]ICYP was also increased in some brain regions of *Tph2*−/− mice. For (***B, C, D***) results are expressed as optical density (OD = specific OD – nonspecific OD) and presented as means ± sem (n = 5). * indicates ANOVA significant output for genotype effect with *p<0.05, **p<0.01, ***p<0.001. For detailed statistical results see Table S1. c: cortex, Ss: somatosensory, Retrospl.: retrosplenial, CA1: cornu ammonis area 1 of hippocampus, g: gyrus, D: dorsal, Enth.: enthorinal, Caud. put.: caudate putamen, V pal.: ventral pallidus, Globus pal.: globus pallidus, Hypo. lat.: lateral hypothalamus, D subic.: dorsal subicullum, Subst. nigra: substantia nigra.

To evaluate the effect of 5-HT deficiency on the regulation of its autoreceptors at the pre- and post-synaptic level, we have quantified 5-HT_1A_ and 5-HT_1B_ receptors in relevant brain regions (Fig. 4).

#### 5-HT_1A_ Receptors

Quantitative autoradiography using the selective 5-HT_1A_ receptor antagonist radioligand [^3^H]WAY 100635 showed that specific labeling of 5-HT_1A_ receptors was increased in *Tph2*−/− compared to *wt* and *Tph2*+/− mice in most of the brain regions tested (Fig. 4*A and 4B*). ANOVA analysis showed a consistent genotype effect (detailed in Table S1) except in retrosplenial and entorhinal cortex. The most significant increases of postsynaptic 5-HT_1A_ heteroceptors compared to *wt* mice were found in the FC and septum (Sep) (+73%; p<0.001 and +63%; p<0.001 respectively), followed by the Hip (+19–22%; 0.04<p<0.001) and amygdala (A) (+15%; p<0.05). In the DR, expressing mostly presynaptic 5-HT_1A_ autoreceptors, the specific labeling was also increased (+12%; p = 0.016) but to a lesser extent than in forebrain regions. The findings from parallel experiments using 5-carboxamidotryptamine (5-CT) to stimulate [^35^S]GTP-γ-S coupling were in accordance with receptor density increases and reflected an enhanced labeling by [^35^S]GTP-γ-S after stimulation in *Tph2*−/− mice particularly in the FC (+54%, p = 0.04 compare to +/+) and Sep (+24%, p = 0.04 compare to *wt*; +/− *vs wt*: +23%, p = 0.048) (Fig. 4*C*, Table S1). This labeling was completely prevented by WAY 100635 showing that it may be specifically attributed to 5-HT_1A_ receptor stimulation. In the brain regions where the increase in 5-HT_1A_ density was more moderate, such as DR, Hip and other cortical regions, the difference in [^35^S]GTP-γ-S coupling did not reach significance.

#### 5-HT_1B_ Receptors

Quantitative autoradiography with iodocyanopindolol ([^125^I]ICYP) in presence of isoproterenol (to mask ß-adrenergic binding sites) showed labeling exclusively in the brain regions with high expression of 5-HT_1B_ receptors. In *Tph2* mutant mice, a significant increase of 5-HT_1B_ receptors labeling was observed in the Sep (−/− *vs wt*: +64%, p = 0.002; −/− *vs* +/−: +30%, p = 0.048), FC (−/− *vs wt*: +63%, p = 0.034), caudate putamen (−/− *vs wt*: +44%, p = 0.034), globus pallidus (−/− *vs wt*: +28%, p = 0.004) and lateral hypothalamus (−/− *vs wt*: +39%, p = 0.025) but not in SN, Hip or DR (Fig. 4*D*, Table S1). [^35^S]GTP-γ-S binding after stimulation did not reveal significant genotype effect but a trend in the SN (F_(2,12)_ = 3.32; p = 0.08), where +/− and −/− mice tend to have lower 5-HT_1B_ stimulation than *wt* (−17% and −14%, respectively), however, between groups comparison did not yield significant differences. Receptor labeling was blocked by the selective 5-HT_1B/1D_ antagonist GR127935. These results demonstrate that, despite the absence of 5-HT synthesis, the expression of these receptors is retained in *Tph2*−/− mice, while they show generalized up-regulation as an adaptation to the lack of endogenous ligand.

### Electrophysiological Properties of Serotonergic Neurons are Preserved in *Tph2*−/− Mice

**Figure 5:**
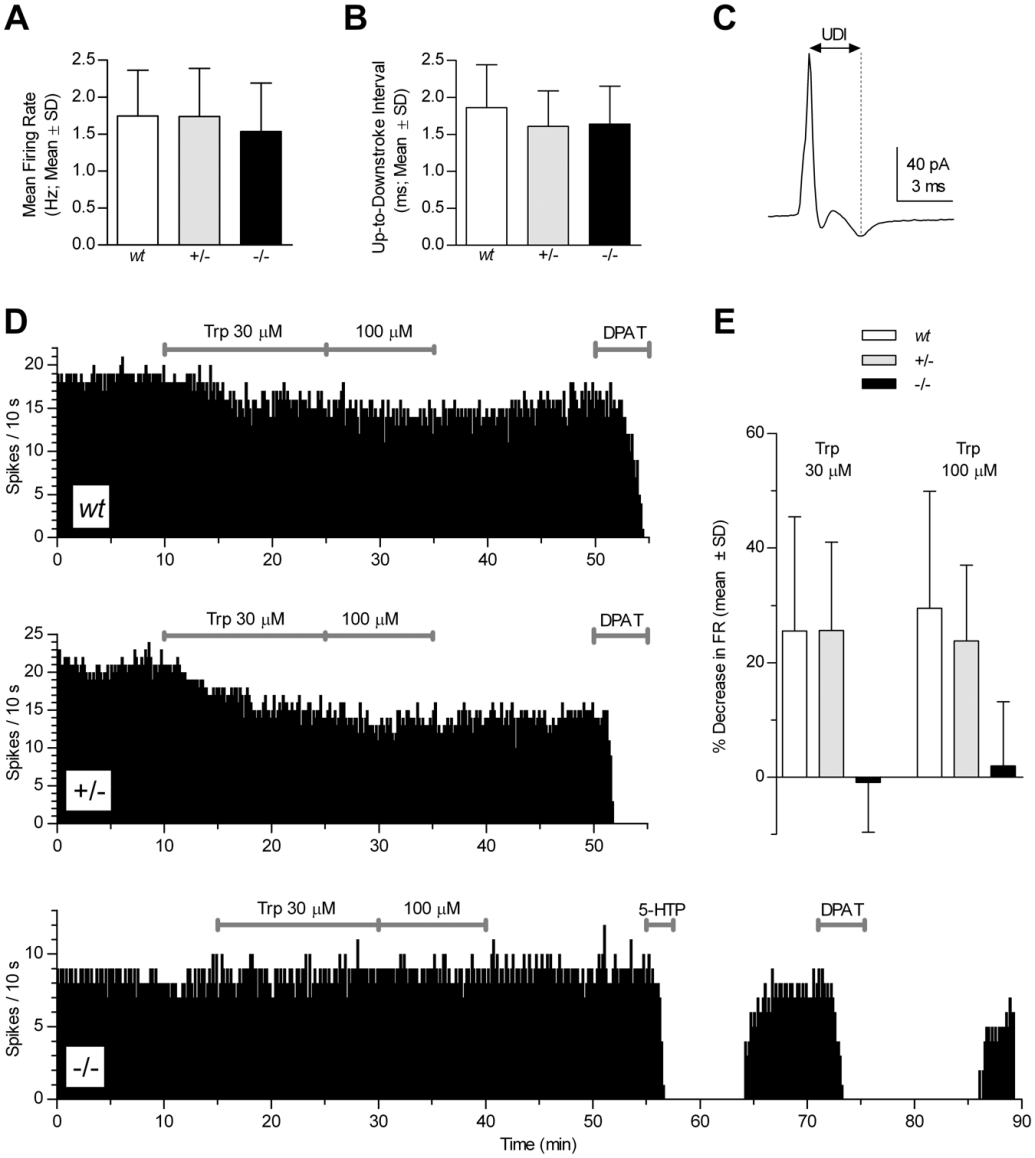
Electrophysiological characteristics of serotonergic raphe neurons. *Tph2*−/− mice displayed electrophysiological characteristics similar to +/− and *wt* mice comprising (***A***) mean firing rate of the recorded neurons measured over a 3 min interval; (***B***) Up-to-Downstroke Interval (UDI) measured as shown in (***C***); *wt*: n = 19; *Tph2*+/−: n = 25; *Tph2*−/−: n = 21. (***D***) Representative time-course of the effect of tryptophan (Trp 30 and 100 µM) and R-8-OH-DPAT (DPAT; 30 nM) application on the firing rate of serotonergic neurons in slices taken from *wt* (upper panel), *Tph2*+/− (middle panel) and *Tph2*−/− (lower panel) mice. Lower panel also illustrates the response of *Tph2*−/− mice to the application of 30 µM L-5-hydroxytryptophan (5-HTP). 5-HTP stopped the firing of serotonergic neurons in all three genotypes. (***E***) Bar graph summarizes the responses to Trp application shown in (***D***). Both concentrations of Trp (30 and 100 µM) did not change firing of serotonergic neurons in *Tph2*−/−, but significantly inhibited serotonergic neuron firing in *wt* and *Tph2+/−* mice (p<0.05, Wilcoxon Signed Rank Test). When compared across genotypes, the responses of *Tph2*−/− serotonergic neurons were statistically different from those of +/− and *wt* mice both for Trp 30 µM (H_(2)_ = 16.28, p<0.0003, *wt* n = 7; *Tph2*+/− n = 10; *Tph2*−/− n = 12) and 100 µM (H_(2)_ = 10.43, p = 0.0054, *wt* n = 7; *Tph2*+/− n = 8; *Tph2*−/− n = 8; Kruskal-Wallis, followed by Dunn’s multiple comparison test). Diagram bars represent means ± SD.

Another critical feature of maturation and physiological function of a specific neuron population is the acquisition of its specific electrophysiological characteristics. To investigate whether Tph2 and thus 5-HT synthesis are required for the development and maintenance of 5-HT neuron-specific electrophysiological activity, we studied serotonergic DR neurons and recorded their spontaneous firing and response to various compounds in order to verify the complete absence of Tph activity and the functionality of autoinhibitory mechanisms (Fig. 5).

#### Activity of 5-HT Devoid Raphe Neurons at Baseline

Serotonergic DR neurons recorded with loose-seal cell-attached voltage clamp in slices taken from *Tph2*−/− and *Tph2*+/− mice showed electrophysiological characteristics similar to those observed in serotonergic neurons of *wt* mice. In the presence of 10 µM phenylephrine, the population of serotonergic neurons recorded from *Tph2*−/− (n = 21), *Tph2*+/− (n = 25) and *wt* (n = 19) mice showed regular firing with similar mean firing rate (F_(2,62)_ = 0.7480, p = 0.4775; Fig. 5*A*). The up-to-downstroke interval of the action current (proportional to action potential half-width, Fig. 5*C*) was also similar across genotypes (F_(2,62)_ = 1.452, p = 0.2421; Fig. 5*B*).

In all recorded neurons from mutant and *wt* mice, application of 30 nM R-8-OH-DPAT (DPAT, 5-HT_1A_ agonist) inhibited firing, indicating typical 5-HT_1A_ autoreceptor function (e.g. Fig. 5*D*).

#### Testing 5-HT Synthesis by a Functional Assay

In slices, application of Trp increases *de novo* synthesis of 5-HT leading to increase in extracellular 5-HT which, in turn, activates somatodendritic 5-HT_1A_ receptors thereby inhibiting serotonergic neuron activity [16]–[18]. To functionally test whether in *Tph2*−/− mice serotonergic neurons are capable to synthesize 5-HT, we studied the effect of the application of 30 and 100 µM Trp on the firing rate of serotonergic neurons. As shown in Fig. 5*D and E*, the superfusion of Trp decreased firing rate of serotonergic neurons recorded in DR slices from *wt* and *Tph2*+/− mice, but not of those from *Tph2*−/− mice, confirming the absence of Tph activity and complete loss of 5-HT synthesis capacity from Trp in these neurons. In addition, in *Tph2*−/− mice, application of 5-HTP (the Tph2 product and 5-HT precursor) reversibly silenced serotonergic neurons (Fig. 5*D*), indicating that 5-HT metabolism downstream of Tph2 and functional response of serotonergic neurons to endogenous 5-HT, when present, are preserved in *Tph2*−/− mice.

## Discussion

Our results provide evidence that gene-targeted *Tph2* inactivation results in 1) loss of brain 5-HT synthesis, 2) growth retardation and persistent leanness but differential age-, sex- and dose-dependent effects on body weight across the lifespan, 3) conserved expression of all known serotonergic neuron-specific markers (except Tph2 and 5-HT), 4) preserved electrophysiological properties characteristic for 5-HT neurons, 5) up-regulation of 5-HT_1A_ and 5-HT_1B_ receptors across brain regions and 6) a reduction of NE concentrations which is consistent with the reduced number of noradrenergic neurons.

### Growth Retardation and Dual Dose-dependent Body Weight Regulation

While a normal overall life expectancy was observed for adult Tph2-deficient mice, both male and female *Tph2−/−* mice consistently displayed leanness with lower body weights than their littermates. Hypomorphism was observed during the early developmental period and persisted across the entire lifespan reflecting reduced fat storage. This reduction of body weight may result from altered regulation at different levels, including reduced food intake, implicating impaired perception of energy needs and satiety; increased metabolic activity and energy expenditure or lower storage, implicating dysregulated glucose, lipid and protein metabolic cycles or thermoregulation. Growth retardation in another mouse line of *Tph2*−/− mice was previously observed but with a catch-up starting after weaning and normal weight reached at age 4 months [19]; however, these mice differ from ours by their genetic background and older mice were not studied. Consistent with our findings, other authors reported persistent low body weight in *Tph2*−/− mice at 24 and 48 weeks associated with a reduced fat pad and size and with both reduced food intake and increased metabolism linked to altered leptin regulation [20].

The leanness of 5-HT deficient mice was unexpected in the face of reports that 5-HT or drugs increasing its release are classically found anorexigenic via hypothalamic actions [21], reducing meal size and body weight [21]–[25] and increasing energy expenditure [26]. While the low body weight of *Tph2*−/− contrasts previous findings and conclusions, observation of obesity in *Tph2*+/− (exhibiting reduced brain 5-HT) as reflected by excess abdominal and pericardial fat storage, particularly in *Tph2*+/− females beyond 24 weeks, concurs. Similar complex dual roles of 5-HT was suggested by studies in the nematode *C. elegans* [27]. In mice, 5-HT transporter null mutants (*5-Htt*−/−), which display increased synaptic 5-HT but a reduced synthesis and total 5-HT brain concentrations [28] also develop obesity in adulthood [29], [30]. In summary, the sex- and gene dose-dependent divergent body weight and fat storage phenotypes in *Tph2* mutant mice supports the notion of a nonlinear dual effect of central 5-HT on somatic development, long-term body weight regulation, and metabolic homeostasis via different pathways interacting with hormonal status.

### 5-HT Deficiency and Impact on other Neurotransmitter Systems

In *Tph2*−/− mutants 5-HT concentrations are dramatically reduced across brain regions and virtually absent from the serotonergic neuron-containing raphe region with only traces detectable by HPLC, demonstrating that 5-HT synthesis within neurons depends on the activity of the Tph2 isoform. While perfusion of brain with removal of most of the residual blood in capillaries resulted in minimal amounts of 5-HT in rostral raphe region at the lower detection limit (<1.2% in *Tph2−/−*), it is still likely that a few blood cells with high 5-HT content, such as platelets or mastocytes, remain trapped in capillaries or that blood diffused post-mortem in brain tissue. Very low brain 5-HT levels were also detected in other *Tph2*−/− mice [19] as well as in *Tph1*/*Tph2−/−* double-knockout mice [31]. In addition, we previously showed that Tph1 is not upregulated in *Tph2*−/− brain indicating that Tph1-driven synthesis can be ruled out in the brain [7]. However, there are several alternative explanations for the remaining traces: 1) HPLC does not detect 5-HT in Tph2-deficient mice but a closely related compound with the same retention time, a possibility which could be resolved by mass spectrometry, 2) minimal amount of the immediate 5-HT precursor, 5-HTP, produced by peripheral Tph1 crosses the blood brain barrier and can be transformed into 5-HT since AADC is ubiquitously expressed, 3) other enzymes, such as phenylalanine hydroxylase, or as yet unknown enzymes, use Trp as substrate and produce a small amount of 5-HT, 4) alternative metabolic pathways are able to produce 5-HT as end- or by-product. Of note, 5-HIAA is more reduced, or even undetectable, than 5-HT itself suggesting that either the metabolic pathway of 5-HT is inhibited, with MAOA activity specifically down-regulated in 5-HT neurons, or the 5-HT-like traces do not represent 5-HT but another compound degraded via another pathway. Taken together, the deficiency in 5-HT availability is so extreme that we assume that 5-HT neurotransmission is abolished in *Tph2*−/− brain despite the presence of neurons with serotonergic cell-like specification.

While DA concentrations are only reduced in Hip, 5-HT deficiency is accompanied by a consistent reduction of NE across brain regions. *Tph2*−/− mice exhibited a reduced number of TH expressing cells in some subparts of the LC which can partly explain the lower NE content in its projection areas. The LC is extensively innervated by Sert-positive fibers containing 5-HT in *wt* controls and devoid of 5-HT in *Tph2−/−* mice. We hypothesize that the absent trophic effect of 5-HT in *Tph2*−/− mice impacts development or survival of NE-specific neurons. Alternatively, absence of 5-HT release prevents the stimulation or inhibits, presumably by indirect input from inhibitory GABAergic or excitatory glutamatergic neurons, expression and activity of TH in NE neurons. Several studies reported that chronic treatment with the SSRI fluoxetine induces an increase of TH gene expression in the LC [32]. Conversely, 5-HT deficiency may thus down-regulate TH activity in the LC, eventually reducing NE biosynthesis. TH is also present along NE fibers projecting towards target areas and regulation at the level of terminals is likely since the DR does not seem to exert a direct inhibitory influence on the release of NE in the LC [33]. While 5-HT and NE fibers with synaptic varicosities colocalize in forebrain regions, a feedback loop involving alpha-2 adrenergic receptors on 5-HT fibers and 5-HT_3_ receptors on NE fibers, allows a reciprocal regulation of release of both neurotransmitters by which 5-HT_3_ receptors stimulate the synaptic release of NE [34]. The stimulation of the neurotransmitter release is accompanied by an activation of its synthesis, whereas the lack of stimulating effect by 5-HT on NE fibers dampens TH activity and thus NE synthesis. The interaction between serotonergic and noradrenergic systems have attracted attention as both systems are implicated in the control of a wide range of complex behaviors as well as the pathogenesis of affective disorders and their treatment by dual 5-HT/NE reuptake inhibitors (SNRI) [35]. Although moderate increase of brain NE content was observed in other *Tph2−/−* mice [20], the NE concentrations reported in [19] and [31] tend to be reduced but the difference did not reach significance. Overall, the findings confirm that serotonergic and noradrenergic systems are interdependent and subject to co-regulation involved in behavior and psychopathology.

### Molecular Specification of Raphe Neurons Lacking 5-HT Synthesis

Although considerable evidence supports morphogenetic properties of 5-HT [3], [4] regulating proliferation, migration and differentiation of neural cells, we did not observe gross neuroanatomical alteration in the brain of *Tph2*−/− mice. One of the aims of the present study was to elucidate whether expression of genes specifying a serotonergic phenotype is conserved in raphe neurons lacking 5-HT synthesis. We demonstrated that Sert is present on the soma of raphe neurons as well as on their fibers and terminals in the various projection areas, although they had lost the capacity to synthesize and thus release 5-HT (Fig. 3 and [5]). In addition to Sert, the serotonergic cell-specific transcription factor Pet1, the monoaminergic-specific Vmat2 as well as 5-HT_1A_ functioning as somatodendritic autoreceptors, are expressed by neurons displaying a 5-HT neuron-like morphological phenotype in *Tph2*−/− mice. Finally, the 5-HT devoid neurons exhibit typical electrophysiological properties of pacemaker firing and are able to produce 5-HT via AADC if supplemented with 5-HTP, suggesting a functional 5-HT synthesis pathway downstream Tph2. While the genetic inactivation of the upstream transcription factors Lmx1b and Pet1 compromises the development of the majority of 5-HT neurons [11]–[13], we conclude that intrinsic 5-HT production is neither essential for the development, differenciation, maintenance and survival of serotonergic neurons, nor for the molecular specification of a serotonergic-like phenotype. It remains, however, to be elucidated in detail whether subtle alteration in dendritic arborization, neurite target finding, or brain structures innervation occurs and whether serotonergic neurons use neuropeptides and/or other monoamines with low affinity for the Sert as physiological or “borrowed” neuromodulator or transmitter in establishing function and connectivity.

### Adaptive 5-HT_1A_ and 5-HT_1B_ Receptors Regulation

The density of 5-HT_1A_ and 5-HT_1B_ receptors and their G-protein coupling were significantly increased across many brain regions of 5-HT deficient *Tph2*−/− male mice, particularly in terminal fields of the FC and Sep. These findings are in accordance with an early study showing that complete abolition of 5-HT synthesis by *p*-chlorophenylalanine (PCPA) treatment led to significant up-regulation of 5-HT_1A_ and 5-HT_1B_ receptor binding sites evaluated in cerebral cortex areas [36]. The opposite phenomenon was observed in mouse models characterized by robust increases of extracellular 5-HT in the brain such as MAO-A null mutant mice where 5-HT_1A_ and 5-HT_1B_ receptors are desensitized and down-regulated [37], [38] and, to a lesser extent and in a brain region specific manner, in *5-Htt*−/− mice [39], [40]. Interestingly, 5-HT_1A_ receptors are down-regulated in patients with depression and anxiety disorders as well as during SSRI treatment [41]–[43]. Sensitization and up-regulation of 5-HT_1A_ and 5-HT_1B_ receptors in 5-HT deficient mice may likely be due to direct cellular and molecular mechanisms compensating for reduced 5-HT ligand availability by an increase of *Htr1a* and *Htr1b* gene expression in target neurons, resulting in increased receptors production and thus increased binding site densities.

### 5-HT Deficient Neurons Retain their Electrophysiological Properties

In *Tph2*−/− mice, serotonergic raphe neurons appear morphologically conserved and express all known markers of serotonergic specification (except Tph2 and 5-HT). In brainstem slices obtained from *Tph2*−/− mice, serotonergic neurons also retained the typical slow (1–2 spikes/s) tonic firing pattern which, together with the preserved shape of the action current, indicates that this spontaneous firing is independent from endogenous 5-HT synthesis and moreover that the absence of 5-HT did not produce adaptive changes of voltage-sensitive membrane channels responsible for the pacemaker activity. Relevant to the functional effectiveness of *Tph2* gene deletion, the fact that Trp did not inhibit the firing of serotonergic neurons clearly shows that synthesis of neuronal 5-HT is mediated exclusively by the Tph2 isoform and that it is abolished in the neurons of *Tph2*−/− mice. However, when in slices from *Tph2−/−* mice, the Tph2-dependent step of 5-HT synthesis is bypassed by supplementation with intermediary 5-HTP (that is converted into 5-HT by AADC) a robust 5-HT_1A_ autoreceptor-mediated inhibition of neuron firing is revealed, showing that responsiveness of serotonergic neurons to 5-HT persists in *Tph2−/−* neurons. This finding, together with the preserved response to the selective 5-HT_1A_ agonist DPAT, shows that 5-HT_1A_ receptors are functional and confirms that the lack of response of serotonergic neurons to Trp in *Tph2−/−* mice is indeed due to the lack of Tph2 and not to the absence of functional 5-HT_1A_-mediated autoinhibition or their downstream effectors. In brainstem slices from *Tph2*+/− mice, serotonergic neurons responded to Trp application with a decrease in firing rate that was similar to that observed in *wt* mice, showing that gene dose dependent reduction of 5-HT synthesis does not result in functional changes in the 5-HT system at baseline. Overall, electrophysiological data provide evidence that endogenous 5-HT is not required for acquisition and preservation of the functional properties typical of serotonergic neurons.

### Conclusion and Perspectives

This study examined the consequence of brain 5-HT deficiency from the earliest stage of ontogeny on somatic growth, brain development and the differentiation of the serotonergic system itself at various levels. First, we found that brain 5-HT has a dose-dependent dual nonlinear effect on the regulation of body weight probably involving distinct mechanisms interacting with hormonal system. Although, *in vitro* and *in vivo* previous studies, using neurotoxins, SSRIs or genetically driven 5-HT alterations [3], [4], [44], indicated a morphogenetic developmental role for 5-HT during brain development, *Tph2*−/− mice are viable and their brain appears structurally normal except a moderate reduction of NE neurons number. Since the mice investigated here were all born to *Tph2*+/− mothers with limited 5-HT loss in the brain (and normal level in the periphery), and the placenta is able to produce 5-HT (e.g. [45]), it is possible that *Tph2*−/− embryos’ brain received during early prenatal development and until their blood brain barrier (BBB) is closed, sufficient 5-HT of exogenous origin (peripheral, placental or maternal) to pursue virtually normal prenatal development, however, a complete compensation is still unlikely. The critical developmental window during which 5-HT signaling deficiency have deleterious effects on brain development is thus in the early phase of development and is independent of endogenous neuronal 5-HT synthesis. Given that Tph2, but nether Tph1, is expressed in the mouse brain from embryonic day (E) 10–10.5 [7], [46], the critical period might be likely before E10, however, a role for exogenous 5-HT is still possible until the BBB is closed. This underlines that, if 5-HT is necessary for normal brain development, exogenous 5-HT, most likely Tph1-derived and of maternal, placental and/or peripheral origin, is the critical source for early prenatal brain development [46]. Alternatively, it seems that, except for the use of neurotoxins which might have other 5-HT-independent toxic side-effects, an excess of 5-HT signaling (in *5-Htt*−/− or SSRI treatments) has more deleterious effects on brain development than 5-HT deficiency. Nevertheless, in adult age, 5-HT synthesis deficiency results in behavioral alterations including in response to environmental stressors (Gutknecht et al., unpublished data), reduces the function of other monoaminergic systems and may also have an impact on brain repair in case of injury. Our results also demonstrate that the proper cellular and molecular differentiation and the maintenance of the serotonergic neuron phenotype do not require endogenous 5-HT synthesis. Moreover, serotonergic neurons continue to display characteristic spontaneous firing although they are devoid of 5-HT showing that this electrophysiological mechanism depends on programming independent from 5-HT synthesis and release. Finally, it remains to be determined whether serotonergic neurons play a physiologically relevant role independent from 5-HT neurotransmission and via which pathway(s). Such supplementary signaling would equip serotonergic neurons with an as yet unknown parallel function.

## Materials and Methods

### Animals and Ethics Statement

All animal manipulations were approved by the review board of the government of lower Franconia and the University of Wuerzburg, and performed according to the European Community guidelines for animal care (Permit number: DL 116/92, application of the European Communities Council Directive 86/609/EEC). A maximum effort was made to minimize the number of animals used and their suffering - see also Supporting Information (SI) (Text S1, Supplemental Materials and Methods). The generation and genotyping procedure of *Tph2*−/− animals have been described in [5]. Their genetic background is composed theoretically of 97% C57BL/6N and 3% Sv129/Ola.

### Body Weight Across the Lifespan

Body weight was determined in different animals at different ages from 3 weeks up to 2.2 years. The weighted cohort included 421 males: 155 *wt*, 173+/− and 93−/− and 374 females: 147 *wt*, 146+/− and 81−/−. Age was used as covariable in the ANOVA comparison between genotypes within each sex.

### Brain Neurotransmitters Concentrations

Two independent cohorts of 4 months old mice were used. The first one was composed of 8 *Tph2*−/− (mixed 4 males and 4 females because of the low number of −/− animals at that time), 8+/− males and 8* wt* males which were perfused for 10 min with PBS. Since no sex effect could be observed, *Tph2*−/− mice were subsequently pooled in the graphs and analyses. ANOVA requirements failed in a majority of cases, therefore, we applied to all non-parametric analysis of variance. Because the efficiency of the brain perfusion was not ideal and blood traces were still visible in the brain of the first animals, a second cohort composed exclusively of males with 4 *Tph2*−/−, 4+/− and 4 *wt*, was used to reanalyze 5-HT concentrations in brain regions. These animals were this time perfused for 10 min at higher pump debit with PBS containing 20 U/ml Heparin. Perfusion was obviously better and brains were visibly whiter. For both cohorts, brains were immediately frozen until brain regions were dissected and neurotransmitters concentrations analysed using HPLC as described in SI (Text S1).

### Histological Staining

Immunohistochemical stainings were performed on brains fixed by perfusion with 4% paraformaldehyde, cryoprotected, frozen and sliced into 14 µm sections. After epitope retrieval and blocking, the primary antibodies against Tph2, Sert and TH were applied, followed by incubation with biotinylated secondary antibodies. Staining was revealed using the Avidin-Biotin Complex method with diaminobenzidine as chromogene. Double-fluorescent 5-HT and Vmat2 primary antibodies immunostaining were realized by applying fluorescent Alexa fluor 488- and Dylight-conjugated secondary antibodies respectively. For *Pet-1 in situ* hybridization, 16 µm sections from native frozen brains were used. Digoxigenin (DIG) labeled *Pet-1* cRNA probes were applied to brain sections and visualized by alkaline phosphatase conjugated anti-DIG antibody. The number of TH-immunoreactive cells was quantified from 8 mice of each genotype. Detailed protocols are described in SI (Text S1).

### Receptors Binding and Stimulation

A cohort of n = 5 males per genotype, 5 months old, were used. Frozen brains were entirely sectioned at 16 µm and spread in 8 adjacent sections series for 5-HT_1A_ and 5-HT_1B_ receptors specific binding and stimulation experiments, as well as the respective non-specific controls.

Specific binding to 5-HT_1A_ was performed with 2 nM [^3^H]-WAY100635 and with 12 pM [^125^I]-Cyanopindolol for 5-HT_1B_. For both receptors, nonspecific binding was estimated from adjacent sections incubated in the same medium supplemented with 10 µM 5-HT. Results are expressed as specific binding OD - nonspecific OD. Agonist-stimulated binding of 0.05 nM [^35^S]-GTP-γ-S was performed with 10^−7 ^M 5-CT. Nonspecific binding was determined from adjacent sections in presence of 1 nM of antagonist WAY100635 for 5-HT_1A_ or GR127935 for 5-HT_1B_.

### Electrophysiological Recording of Raphe Neurons

Mice (28 to 80 days old) were anaesthetized with isofluorane and decapitated. The brain was rapidly removed, dissected in ice-cold gassed (95% O_2_ and 5% CO_2_) artificial cerebrospinal fluid (ACSF) containing (in mM): 124 NaCl, 2.75 KCl, 1.25 NaH_2_PO_4_, 1.3 MgCl_2_, 2 CaCl_2_, 26 NaHCO_3_, 11 D-glucose (pH 7.4), and the brainstem was sliced coronally into 200 µm thick slices with a vibratome. After recovery, the slices were individually transferred into the recording chamber and superfused continuously with warmed ACSF (34–35°C) at a rate of 2 ml min^−1^. Neurons were visualized by infrared differential interference contrast video microscopy with a Newicon C2400-07 camera (Hamamatsu, Hamamatsu City, Japan) mounted to an Axioskop microscope (Zeiss, Göttingen, Germany). Recordings were made using an EPC-10 amplifier (HEKA Elektronic, Lamberecht, Germany). Patch pipettes were prepared from thick-walled borosilicate glass on a P-97 Brown-Flaming electrode puller (Sutter Instruments, Novato, CA) and had resistance of 3–6 MΩ when filled with solution containing (in mM): 125 NaCl, 10 HEPES, 2.75 KCl, 2 CaCl_2_, 1.3 MgCl_2_ (pH 7.4 with NaOH). Loose-seal cell-attached recordings (5–20 MΩ seal resistance) were acquired continuously in voltage-clamp mode. Signals were filtered at 3 kHz and digitized at 10 kHz. Pipette potential was maintained at 0 mV. Recordings were aborted if firing rate was sensitive to changes in pipette holding potential or if shape of action current changed. Data were analyzed using Clampfit 9.2 (Molecular Devices) and Prism 5 (GraphPad Software, San Diego, CA). Extracellular saline was supplemented with 10 µM phenylephrine to facilitate firing. Neurons were presumed serotonergic when 1) displayed firing rate of less than 3.5 Hz, 2) had asymmetric action current with peak-to-peak interval greater than 1 ms, and 3) their firing stopped in response to application of the 5-HT_1A_ receptor agonist DPAT (30 nM) at the end of experiment. Since experiments depended on endogenous 5-HT, recordings were done from neurons located at least 50 µm below the slice surface [18]. One experiment was done in each slice. The number of used mice and recorded cells for each particular design is indicated in results section and Fig. 5 legend.

### Statistical Analysis

Unless otherwise specified, such as for electrophysiological data analysis (see Figure legend), the effects of genotype and sex were analyzed using ANOVA (indicated by F_(df1,df2)_ values) followed up with Tuckey-HSD post hoc tests for multiple group comparison. When requirements for one-way ANOVA (normal distribution, equality of variances) were not fulfilled, non-parametric Kruskall-Wallis analysis of variance was applied (indicated by H_(df)_ values), followed by Mann-Whitney-U-Test. P<0.05 was considered statistically significant and 0.05<p≤0.10 was considered as a trend of significance.

## Supporting Information

Table S1 **Detailed statistical results of the genotype effect in the analysis of variance and post hoc tests for 5-HT_1A_ and 5-HT_1B_ receptor binding densities and 5-HT_1A_ GTP-γ-S coupling in various brain regions, n = 5 males.** nd: non-determined, ns: non-significant.
(DOCX)

Text S1 **Supplemental Materials and Methods.**
(DOCX)

## References

[1] Gross C, Hen R (2004) The developmental origins of anxiety. Nat Rev Neurosci 5: 545–552. 15208696 10.1038/nrn1429

[2] Heiming RS, Jansen F, Lewejohann L, Kaiser S, Schmitt A, et al (2009) Living in a dangerous world: the shaping of behavioral profile by early environment and 5-HTT genotype. Front Behav Neurosci 3: 26. 19826611 10.3389/neuro.08.026.2009 PMC2759357

[3] Gaspar P, Cases O, Maroteaux L (2003) The developmental role of serotonin: news from mouse molecular genetics. Nat Rev Neurosci 4: 1002–1012. 14618156 10.1038/nrn1256

[4] Daubert EA, Condron BG (2010) Serotonin: a regulator of neuronal morphology and circuitry. Trends Neurosci 33: 424–434. 20561690 10.1016/j.tins.2010.05.005 PMC2929308

[5] Gutknecht L, Waider J, Kraft S, Kriegebaum C, Holtmann B, et al (2008) Deficiency of brain 5-HT synthesis but serotonergic neuron formation in Tph2 knockout mice. J Neural Transm 115: 1127–1132. 18665319 10.1007/s00702-008-0096-6

[6] Cote F, Thevenot E, Fligny C, Fromes Y, Darmon M, et al (2003) Disruption of the nonneuronal tph1 gene demonstrates the importance of peripheral serotonin in cardiac function. Proc Natl Acad Sci U S A 100: 13525–13530. 14597720 10.1073/pnas.2233056100 PMC263847

[7] Gutknecht L, Kriegebaum C, Waider J, Schmitt A, Lesch KP (2009) Spatio-temporal expression of tryptophan hydroxylase isoforms in murine and human brain: convergent data from Tph2 knockout mice. Eur Neuropsychopharmacol 19: 266–282. 19181488 10.1016/j.euroneuro.2008.12.005

[8] Walther DJ, Peter JU, Bashammakh S, Hortnagl H, Voits M, et al (2003) Synthesis of serotonin by a second tryptophan hydroxylase isoform. Science 299: 76. 12511643 10.1126/science.1078197

[9] Beaulieu JM, Zhang X, Rodriguiz RM, Sotnikova TD, Cools MJ, et al (2008) Role of GSK3 beta in behavioral abnormalities induced by serotonin deficiency. Proc Natl Acad Sci U S A 105: 1333–1338. 18212115 10.1073/pnas.0711496105 PMC2234138

[10] Jacobsen JP, Siesser WB, Sachs BD, Peterson S, Cools MJ, et al (2011) Deficient serotonin neurotransmission and depression-like serotonin biomarker alterations in tryptophan hydroxylase 2 (Tph2) loss-of-function mice. Mol Psychiatry May 3. 10.1038/mp.2011.50 PMC3536482 21537332

[11] Hendricks TJ, Fyodorov DV, Wegman LJ, Lelutiu NB, Pehek EA, et al (2003) Pet-1 ETS gene plays a critical role in 5-HT neuron development and is required for normal anxiety-like and aggressive behavior. Neuron 37: 233–247. 12546819 10.1016/s0896-6273(02)01167-4

[12] Ding YQ, Marklund U, Yuan W, Yin J, Wegman L, et al (2003) Lmx1b is essential for the development of serotonergic neurons. Nat Neurosci 6: 933–938. 12897786 10.1038/nn1104

[13] Song NN, Xiu JB, Huang Y, Chen JY, Zhang L, et al (2011) Adult raphe-specific deletion of Lmx1b leads to central serotonin deficiency. PLoS One 6: e15998. 21246047 10.1371/journal.pone.0015998 PMC3016403

[14] Kiyasova V, Fernandez SP, Laine J, Stankovski L, Muzerelle A, et al (2011) A Genetically Defined Morphologically and Functionally Unique Subset of 5-HT Neurons in the Mouse Raphe Nuclei. J Neurosci 31: 2756–2768. 21414898 10.1523/JNEUROSCI.4080-10.2011 PMC6623784

[15] Zhao ZQ, Scott M, Chiechio S, Wang JS, Renner KJ, et al (2006) Lmx1b is required for maintenance of central serotonergic neurons and mice lacking central serotonergic system exhibit normal locomotor activity. J Neurosci 26: 12781–12788. 17151281 10.1523/JNEUROSCI.4143-06.2006 PMC6674835

[16] Audero E, Coppi E, Mlinar B, Rossetti T, Caprioli A, et al (2008) Sporadic autonomic dysregulation and death associated with excessive serotonin autoinhibition. Science 321: 130–133. 18599790 10.1126/science.1157871

[17] Gallager DW, Aghajanian GK (1976) Inhibition of firing of raphe neurones by tryptophan and 5-hydroxytryptophan: blockade by inhibiting serotonin synthesis with Ro-4-4602. Neuropharmacology 15: 149–156. 1084488 10.1016/0028-3908(76)90023-x

[18] Mlinar B, Tatini F, Ballini C, Nencioni S, Della Corte L, et al (2005) Differential autoinhibition of 5-hydroxytryptamine neurons by 5-hydroxytryptamine in the dorsal raphe nucleus. Neuroreport 16: 1351–1355. 16056138 10.1097/01.wnr.0000175249.25535.bf

[19] Alenina N, Kikic D, Todiras M, Mosienko V, Qadri F, et al (2009) Growth retardation and altered autonomic control in mice lacking brain serotonin. Proc Natl Acad Sci U S A 106: 10332–10337. 19520831 10.1073/pnas.0810793106 PMC2700938

[20] Yadav VK, Oury F, Suda N, Liu ZW, Gao XB, et al (2009) A serotonin-dependent mechanism explains the leptin regulation of bone mass, appetite, and energy expenditure. Cell 138: 976–989. 19737523 10.1016/j.cell.2009.06.051 PMC2768582

[21] Leibowitz SF, Alexander JT (1998) Hypothalamic serotonin in control of eating behavior, meal size, and body weight. Biol Psychiatry 44: 851–864. 9807640 10.1016/s0006-3223(98)00186-3

[22] Conductier G, Crosson C, Hen R, Bockaert J, Compan V (2005) 3,4-N-methlenedioxymethamphetamine-induced hypophagia is maintained in 5-HT1B receptor knockout mice, but suppressed by the 5-HT2C receptor antagonist RS102221. Neuropsychopharmacology 30: 1056–1063. 15668722 10.1038/sj.npp.1300662

[23] Jean A, Conductier G, Manrique C, Bouras C, Berta P, et al (2007) Anorexia induced by activation of serotonin 5-HT4 receptors is mediated by increases in CART in the nucleus accumbens. Proc Natl Acad Sci U S A 104: 16335–16340. 17913892 10.1073/pnas.0701471104 PMC2042207

[24] Curzon G (1990) Serotonin and appetite. Ann N Y Acad Sci 600: 521–530; discussion 530–521. 2252331 10.1111/j.1749-6632.1990.tb16907.x

[25] Meguid MM, Fetissov SO, Varma M, Sato T, Zhang L, et al (2000) Hypothalamic dopamine and serotonin in the regulation of food intake. Nutrition 16: 843–857. 11054589 10.1016/s0899-9007(00)00449-4

[26] Bross R, Hoffer LJ (1995) Fluoxetine increases resting energy expenditure and basal body temperature in humans. Am J Clin Nutr 61: 1020–1025. 7733022 10.1093/ajcn/61.4.1020

[27] Srinivasan S, Sadegh L, Elle IC, Christensen AG, Faergeman NJ, et al (2008) Serotonin regulates C. elegans fat and feeding through independent molecular mechanisms. Cell Metab 7: 533–544. 18522834 10.1016/j.cmet.2008.04.012 PMC2495008

[28] Murphy DL, Lesch KP (2008) Targeting the murine serotonin transporter: insights into human neurobiology. Nat Rev Neurosci 9: 85–96. 18209729 10.1038/nrn2284

[29] Erritzoe D, Frokjaer VG, Haahr MT, Kalbitzer J, Svarer C, et al (2010) Cerebral serotonin transporter binding is inversely related to body mass index. Neuroimage 52: 284–289. 20382236 10.1016/j.neuroimage.2010.03.086

[30] Uceyler N, Schutt M, Palm F, Vogel C, Meier M, et al (2010) Lack of the serotonin transporter in mice reduces locomotor activity and leads to gender-dependent late onset obesity. Int J Obes (Lond) 34: 701–711. 20084070 10.1038/ijo.2009.289

[31] Savelieva KV, Zhao S, Pogorelov VM, Rajan I, Yang Q, et al (2008) Genetic disruption of both tryptophan hydroxylase genes dramatically reduces serotonin and affects behavior in models sensitive to antidepressants. PLoS One 3: e3301. 18923670 10.1371/journal.pone.0003301 PMC2565062

[32] Brady LS, Gold PW, Herkenham M, Lynn AB, Whitfield HJ Jr (1992) The antidepressants fluoxetine, idazoxan and phenelzine alter corticotropin-releasing hormone and tyrosine hydroxylase mRNA levels in rat brain: therapeutic implications. Brain Res 572: 117–125. 1351783 10.1016/0006-8993(92)90459-m

[33] Pudovkina OL, Cremers TI, Westerink BH (2002) The interaction between the locus coeruleus and dorsal raphe nucleus studied with dual-probe microdialysis. Eur J Pharmacol 445: 37–42. 12065192 10.1016/s0014-2999(02)01663-1

[34] Mongeau R, Blier P, de Montigny C (1997) The serotonergic and noradrenergic systems of the hippocampus: their interactions and the effects of antidepressant treatments. Brain Res Brain Res Rev 23: 145–195. 9164669 10.1016/s0165-0173(96)00017-3

[35] Sziray N, Kuki Z, Nagy KM, Marko B, Kompagne H, et al (2010) Effects of single and simultaneous lesions of serotonergic and noradrenergic pathways on open-space and bright-space anxiety-like behavior in two animal models. Behav Brain Res 209: 93–98. 20096733 10.1016/j.bbr.2010.01.019

[36] Compan V, Segu L, Buhot MC, Daszuta A (1998) Differential effects of serotonin (5-HT) lesions and synthesis blockade on neuropeptide-Y immunoreactivity and 5-HT1A, 5-HT1B/1D and 5-HT2A/2C receptor binding sites in the rat cerebral cortex. Brain Res 795: 264–276. 9622647 10.1016/s0006-8993(98)00316-3

[37] Evrard A, Malagie I, Laporte AM, Boni C, Hanoun N, et al (2002) Altered regulation of the 5-HT system in the brain of MAO-A knock-out mice. Eur J Neurosci 15: 841–851. 11906526 10.1046/j.1460-9568.2002.01917.x

[38] Lanoir J, Hilaire G, Seif I (2006) Reduced density of functional 5-HT1A receptors in the brain, medulla and spinal cord of monoamine oxidase-A knockout mouse neonates. J Comp Neurol 495: 607–623. 16498683 10.1002/cne.20916

[39] Fabre V, Beaufour C, Evrard A, Rioux A, Hanoun N, et al (2000) Altered expression and functions of serotonin 5-HT1A and 5-HT1B receptors in knock-out mice lacking the 5-HT transporter. Eur J Neurosci 12: 2299–2310. 10947809 10.1046/j.1460-9568.2000.00126.x

[40] Li Q, Wichems C, Heils A, Lesch KP, Murphy DL (2000) Reduction in the density and expression, but not G-protein coupling, of serotonin receptors (5-HT1A) in 5-HT transporter knock-out mice: gender and brain region differences. J Neurosci 20: 7888–7895. 11050108 10.1523/JNEUROSCI.20-21-07888.2000 PMC6772750

[41] Lesch KP, Gutknecht L (2004) Focus on The 5-HT1A receptor: emerging role of a gene regulatory variant in psychopathology and pharmacogenetics. Int J Neuropsychopharmacol 7: 381–385. 15683551 10.1017/S1461145704004845

[42] Drevets WC, Thase ME, Moses-Kolko EL, Price J, Frank E, et al (2007) Serotonin-1A receptor imaging in recurrent depression: replication and literature review. Nucl Med Biol 34: 865–877. 17921037 10.1016/j.nucmedbio.2007.06.008 PMC2702715

[43] Savitz J, Lucki I, Drevets WC (2009) 5-HT(1A) receptor function in major depressive disorder. Prog Neurobiol 88: 17–31. 19428959 10.1016/j.pneurobio.2009.01.009 PMC2736801

[44] Simpson KL, Weaver KJ, de Villers-Sidani E, Lu JY, Cai Z, et al (2011) Perinatal antidepressant exposure alters cortical network function in rodents. Proc Natl Acad Sci U S A 108: 18465–18470. 22025710 10.1073/pnas.1109353108 PMC3215047

[45] Bonnin A, Goeden N, Chen K, Wilson ML, King J, et al (2011) A transient placental source of serotonin for the fetal forebrain. Nature 472: 347–350. 21512572 10.1038/nature09972 PMC3084180

[46] Cote F, Fligny C, Bayard E, Launay JM, Gershon MD, et al (2007) Maternal serotonin is crucial for murine embryonic development. Proc Natl Acad Sci U S A 104: 329–334. 17182745 10.1073/pnas.0606722104 PMC1713169

