# Supplementary material for: Impacts of Brain Serotonin Deficiency following Tph2 Inactivation on Development and Raphe Neuron Serotonergic Specification
Source: PLoS One. 2012 Aug 17;7(8):e43157. doi: 10.1371/journal.pone.0043157 (PMC3422228; doi:10.1371/journal.pone.0043157)
Supplement: Table S1 — Detailed statistical results of the genotype effect in the analysis of variance and post hoc tests for 5-HT1A and 5-HT1B receptor binding densities and 5-HT1A GTP-γ-S coupling in various brain regions, n = 5 males. nd: non-determined, ns: non-significant. (DOCX) [file pone.0043157.s001.docx]

|  |  | **5-HT_1A_ Binding** | | | **5-HT_1A_ GTP-**γ**-S binding** | |  |  |  | **5-HT_1B_ Binding** | | |
| --- | --- | --- | --- | --- | --- | --- | --- | --- | --- | --- | --- | --- |
| BRAIN REGIONS | Analyses of Variance (p) | | | F _(2,12)_ H_(2)_ | Analyses of Variance (p) | |  | BRAIN REGIONS | Analyses of Variance (p) F _(2,12)_ | | | |
|  |  | | % change | post hoc test | % change | post hoc test |  |  |  | | % change | post hoc test |
|  |  | |  |  |  |  |  |  |  | |  |  |
| **Frontal cortex** | **F=34.88 (<.001)** | | |  | **F=4.0 (.046)** |  |  | **Frontal cortex** | **F=4.2 (.042)** | | |  |
|  | *wt* / *-/-* | | +73.1 | **<.001** | +54.3 | **.04** |  |  | *wt* / *-/-* | | +63.0 | **.034** |
|  | *wt* / *+/-* | | +32.7 | **.008** | +19.3 | ns |  |  | *wt* / *+/-* | | +35.2 | ns |
|  | -/- / +/- | | +30.5 | **.002** | +29.3 | ns |  |  | -/- / +/- | | +20.6 | ns |
|  |  | |  |  |  |  |  |  |  | |  |  |
| **Somatosensori** | **H=6.3 (.042)** | | |  | F=1.15 (ns) |  |  | **Cadate putamen** | **F=4.7 (.032)** | | |  |
| **cortex** | *wt* / *-/-* | | +39.4 | **<.05** |  |  |  |  | *wt* / *-/-* | | +43.6 | **.034** |
|  | *wt* / *+/-* | | +6.7 | ns |  |  |  |  | *wt* / *+/-* | | +8.7 | ns |
|  | -/- / +/- | | +30.7 | ns |  |  |  |  | -/- / +/- | | +32.2 | .093 |
|  |  | |  |  |  |  |  |  |  | |  |  |
| **Septum** | **F=67.4 (<.001)** | | |  | **F=4.99 (.026)** | |  | **Septum** | **F=10.3 (.003)** | | |  |
|  | *wt* / *-/-* | | +63.2 | **<.001** | +23.9 | **.041** |  |  | *wt* / *-/-* | | +63.9 | **.002** |
|  | *wt* / *+/-* | | +49.7 | **<.001** | +22.7 | **.048** |  |  | *wt* / *+/-* | | +25.6 | ns |
|  | -/- / +/- | | +9.1 | .084 | +0.99 | ns |  |  | -/- / +/- | | +30.5 | **.048** |
|  |  | |  |  |  |  |  |  |  | |  |  |
| **CA1** | **F=12.1 (.001)** | | |  | H=1.94 (ns) |  |  | **Ventral pallidus** | F=0.4 (ns) | | |  |
|  | *wt* / *-/-* | | +20.7 | **.001** |  |  |  |  |  | |  |  |
|  | *wt* / *+/-* | | +8.9 | ns |  |  |  | **Globus pallidus** | **F=8.35 (.005)** | | |  |
|  | -/- / +/- | | +10.8 | **.041** |  |  |  |  | *wt* / *-/-* | | +28.5 | **.004** |
|  |  | |  |  |  |  |  |  | *wt* / *+/-* | | +15.1 | ns |
| **Dentate gyrus** | **F=6.7 (.011)** | | |  | nd |  |  |  | -/- / +/- | | +11.7 | ns |
|  | *wt* / *-/-* | | +19.2 | **.04** |  |  |  |  |  | |  |  |
|  | *wt* / *+/-* | | -4.5 | ns |  |  |  | **Lateral hypothalamus** | **F=4.7 (.031)** | | |  |
|  | -/- / +/- | | +24.9 | **.012** |  |  |  |  | *wt* / *-/-* | | +38.8 | **.025** |
|  |  | |  |  |  |  |  |  | *wt* / *+/-* | | +15.9 | ns |
| **Amygdala** | **H=8.0 (.018)** | | |  | nd |  |  |  | -/- / +/- | | +19.6 | ns |
|  | *wt* / *-/-* | | +26.6 | **<.05** |  |  |  |  |  | |  |  |
|  | *wt* / *+/-* | | +0.3 | ns |  |  |  | **CA1** | F=0.76 (ns) | |  |  |
|  | -/- / +/- | | +26.3 | **<.05** |  |  |  |  |  | |  |  |
|  |  | |  |  |  |  |  | **Dorsal subiculum** | F=2.5 (ns) | |  |  |
| **Dorsal raphe** | **F=5.6 (.019)** | | |  | H=2.16 (ns) |  |  |  |  | |  |  |
|  | *wt* / *-/-* | | +11.7 | **.016** |  |  |  | **Substantia nigra** | F=0.17 (ns) | |  |  |
|  | *wt* / *+/-* | | +4.3 | ns |  |  |  |  |  | |  |  |
|  | -/- / +/- | | +7.1 | ns |  |  |  | **Dorsal raphe** | F=0.25 (ns) | |  |  |
|  |  | |  |  |  |  |  |  |  | |  |  |
| **Entorhinal cortex** | F=1.28 (ns) | |  |  | H=1.6 (ns) |  |  | **Entorhinal cortex** | F=0.58 (ns) | |  |  |
|  |  | |  |  |  |  |  |  |  |  |  |  |
| **Retrosplenial cortex** | F=1.3 (ns) | |  |  | nd |  |  |  |  |  |  |  |
