# Supplementary material for: Impacts of Brain Serotonin Deficiency following Tph2 Inactivation on Development and Raphe Neuron Serotonergic Specification
Source: PLoS One. 2012 Aug 17;7(8):e43157. doi: 10.1371/journal.pone.0043157 (PMC3422228; doi:10.1371/journal.pone.0043157)
Supplement: Text S1 — Supplemental Materials and Methods. (DOCX) [file pone.0043157.s002.docx]

**SUPPLEMENTAL MATERIALS AND METHODS**

**General information.** All animal manipulations were approved by the review board of the government of lower Franconia and the University of Wuerzburg, and performed according to the European Community guidelines for animal care (Permit number: DL 116/92, application of the European Communities Council Directive 86/609/EEC). A maximum effort was made to minimize the number of animals used and their suffering. All mice sacrifice, transcardial perfusion or brain collection were realized under deep anesthesia obtained by isoflurane inhalation. Perfusion of the animals occurred through injection for 10 min of the perfusing solution in the left ventricle of the heart using a peristaltic pump. Freezing of brains occurred through immersion in precooled 2-methyl butane on dry ice.

**Measure of neurotransmitters concentrations in brain regions.** For both cohorts, brains were collected after perfusion, immediately frozen and stored at -80°C until use. Brain regions were quickly dissected on a cold plate and refrozen on dry ice until sonicated in ice-cold H_3_PO_4_ (150 mM), DTPA (500 µM) with an ultrasonic probe preset to 15% amplitude and 0.5 sec pulse interval. Samples were centrifuged for 20 min at 4°C and 36000 x g. For the detection of the neurotransmitters Norepinephrine (NE), Dopamine (DA), 5-HT and its metabolite (5-HIAA), supernatants (40 µl each) were injected into an HPLC system (Agilent) consisting of EC 250/4.6 Nucleosil 100-3-C18 reversed-phase columns (Machery-Nagel) and an electrochemical detector (BioRad) which was adjusted to 0.75 V. Composition of mobile phase was 90% 0.65mM octanesulfonic acid, pH 3.51, 10% methanol, 0.5 mM triethylamine, 0.1 mM EDTA and 0.1 M NaH_2_PO_4_.

**Immunohistochemistry (IHC).** Brains were perfused with phosphate buffered saline (PBS) first, and then with 4% paraformaldehyde (PFA) in PBS, pH7.5 for 5 min each. After collection they were post-fixed overnight in 4% PFA, then cryoprotected by successive infiltration with 10 and 20% sucrose (in PBS) and frozen. Subsequently, 14 µm thick cryostat coronal sections were prepared and mounted on glass slides. The staining protocol started with a heat-induced epitope retrieval performed at 96°C for 14 min in 10 mM citrate buffer (pH 6.0) containing 0.05 % Tween 20.

For the detection of Tph2, Sert, and TH, labeling using Avidin-Biotin Complex methods with diaminobenzidine (DAB) as chromogene was performed. First, endogenous peroxidase activity was blocked for 30 min with 0.6% H_2_O_2_ in Tris buffered saline (TBS), sections were then preincubated for 1 h at room temperature (RT) in the blocking solution, containing 5% normal goat serum (NGS), 2% BSA and 0.25% Triton X-100 diluted in TBS, to prevent non-specific binding. Tissue sections were subsequently exposed overnight at 4°C to primary antibodies diluted in the blocking solution: rabbit anti-Tph2 (1:1000, self-made, see (5, 7)), rabbit anti-Sert (1:750, Stratagene), rabbit anti-5-HT (1:10000, Incstar) and mouse anti-TH (1:5000, Chemicon). The next day, tissue sections were incubated with biotinylated secondary antibodies (goat anti-rabbit IgG, 1:900 or goat anti-mouse IgG, 1:5000, both from Vector Laboratories) diluted in 2% NGS, 2% BSA and 0.25% Triton-X100 in TBS for 1.5 h and in streptavidin-biotin-peroxidase complex (Vector Laboratories) for 1.5 h at RT. Peroxidase activity was visualized using DAB substrate (metal enhanced; Roche).

For 5-HT and Vmat2 double-immunofluorescent detection, sections were first preincubated for 1.5 h at RT in blocking solution (5% normal donkey serum; 0.25% Triton X-100 in TBS) to prevent non-specific binding of secondary antibodies. The pairs of primary antibodies rabbit anti-5-HT (Immunostar) and guinea pig anti-vmat2 (Acris) were diluted in blocking solution at 1:14000 and 1:800 respectively and applied to brain slices overnight at 4°C. The next day, tissue sections were incubated with fluorescent secondary antibodies (donkey Alexa Fluor 488-conjugated anti-rabbit, Invitrogen; and donkey Dylight 549-conjugated anti-guinea pig, ImmunoResearch) diluted 1:500 in blocking solution for 1.5 h at RT. After washing with TBS, 300 nM DAPI (Invitrogen) counterstaining was performed for 5 min at RT followed by another wash with TBS and mounting with Gel-Mount (Science Services). Stacks of z-axis images were obtained every 1.5 µm and merged according to middle intensity. Colocalization of the respective immunofluorescent signals for 5-HT vs Vmat2 were visualized by fluorescent signal overlay using a wide-field fluorescence microscope (Olympus BX40) and analysed by Olympus cell P software (Version 2.8).

**TH-immunoreactive cell quantification.** TH-positive cells were examined in 14 µm-thick coronal microtome frozen sections spaced 84 µm apart. Brain regions chosen for quantification included substantia nigra, ventral tegmental area, retrorubal field A8 as well as locus coeruleus. For each region, between 3 and 4 homologous slices were analyzed from 8 mice of each genotype. Counting of cells and determination of surface area occupied by certain cell clusters was accomplished with the help of a Neurolucida microscopy system (MicroBrightField) allowing encircling areas of interest by means of individual contours on both sides of the brain. For all section series, contours were set as equivalent as possible to assure reliable evaluation of surface area and cell density. During the whole procedure, the investigator was kept unaware of the animals’ genotype.

***In situ* hybridization.** *Pet-1* cDNA probe of 491 bp of lenth was generated by PCR from 3´UTR and cloned in pGEMT vector (Promega)*.* The identity of the cDNA insert was verified by sequencing. Following plasmid linearization and purification, sense and antisense digoxigenin (DIG) labeled cRNA probes were generated using the DIG RNA labeling kit (SP6/T7, Roche). The cRNA probes were purified and analyzed on 1% agarose gels.

Hybridization with the *Pet-1* cRNA probe was carried out on 16 µm frozen sections. After fixation for 5 min in freshly prepared cold 4% PFA in 0.1 M PBS and subsequent storage in 100% ethanol for several hours, they were transferred to 2xSSC (sodium saline citrate) through a graded series of ethanol (100%, 95%, 80%, 70% ethanol) and treated with 0.02 N HCl for 5 min. After washing with 2xSSC, sections were incubated with freshly prepared 0.25% acetic anhydride in 0.1 M triethanolamine for at least 20 min, washed again with 2xSSC and covered with the prehybridization solution for 1 h at 60°C. The prehybridization solution contained 50% deionized formamide, 4xSSC, 1x Denhardt’s solution, 10% dextransulfate and 250 µg/ml denatured Salmon sperm DNA. Then, sections were incubated with the hybridization solution containing prehybridization buffer and DIG-labeled antisense (or sense as negative control) cRNA (final concentration 15 ng/µl) for 16-18 h at 60°C.

Posthybridization washes were done stepwise with 2xSSC at RT, 50% formamide in 2xSSC (at 60°C), and then again with 2xSSC. To remove unhybridized single stranded RNAs, sections were treated with 40 µg/ml ribonuclease A (RNase A) in a solution containing 500 mM NaCl, 10 mM Tris-HCl (pH 8.0) and 1 mM EDTA at 37°C for 30 min. After several washes with the same buffer without RNase A at RT, sections were incubated in this buffer at 60°C for 30 min and rinsed in TBS. DIG-labeled cRNA probes were visualized with a sheep anti-DIG alkaline phosphatase (aP) conjugated antibody (1.5 U/ml) and 5-bromo-4-chloro-3-indolyl phosphate (BCIP) and 4-nitro-blue-tetracolium-chloride (NBT), as the substrate of aP (provided by the Nucleic Acid Detection Kit, Roche). Substitution of the antisense cRNA probes by an equivalent amount of labeled sense cRNA or RNase A treatment before hybridization led to a lack of staining. From these findings it was concluded that the antisense probes were specific and DIG detections did not create labelling artefacts.
